# Cladielloides A and B: New Eunicellin-Type Diterpenoids from an Indonesian Octocoral *Cladiella* sp

**DOI:** 10.3390/md8122936

**Published:** 2010-12-06

**Authors:** Yung-Husan Chen, Chia-Ying Tai, Tsong-Long Hwang, Ching-Feng Weng, Jan-Jung Li, Lee-Shing Fang, Wei-Hsien Wang, Yang-Chang Wu, Ping-Jyun Sung

**Affiliations:** 1 National Museum of Marine Biology & Aquarium, Pingtung 944, Taiwan; E-Mails: tony_chen72001@yahoo.com.tw (Y.-H.C.); j19851214@hotmail.com (C.-Y.T.); jj@nmmba.gov.tw (J.-J.L.); whw@nmmba.gov.tw (W.-H.W.); 2 Graduate Institute of Marine Biotechnology, National Dong Hwa University, Pingtung 944, Taiwan; 3 Graduate Institute of Natural Products, Chang Gung University, Taoyuan 333, Taiwan; E-Mail: htl@mail.cgu.edu.tw (T.-L.H.); 4 Department of Life Science and Institute of Biotechnology, National Dong Hwa University, Hualien 974, Taiwan; E-Mail: cfweng@mail.ndhu.edu.tw (C.-F.W.); 5 Department of Sport, Health, and Leisure, Cheng Shiu University, Kaohsiung 833, Taiwan; E-Mail: lsfang@csu.edu.tw (L.-S.F.); 6 Department of Marine Biotechnology and Resources, National Sun Yat-sen University, Kaohsiung 804, Taiwan; 7 Asia-Pacific Ocean Research Center, National Sun Yat-sen University, Kaohsiung 804, Taiwan; 8 Graduate Institute of Integrated Medicine, College of Chinese Medicine, China Medical University, Taichung 404, Taiwan; 9 Natural Medicinal Products Research Center, China Medical University Hospital, Taichung 404, Taiwan

**Keywords:** cladielloide, eunicellin, octocoral, cytotoxicity, superoxide anion, elastase

## Abstract

Two new eunicellin-type diterpenoids, cladielloides A (**1**) and B (**2**), which were found to possess a 2-hydroxybutyroxy group in their structures, were isolated from an Indonesian octocoral identified as *Cladiella* sp. The structures of eunicellins **1** and **2** were elucidated by spectroscopic methods. Cladielloide B (**2**) exhibited moderate cytotoxicity toward CCRF-CEM tumor cells and this compound displayed significant inhibitory effects on superoxide anion generation and elastase release by human neutrophils.

## 1. Introduction

**Scheme 1:**
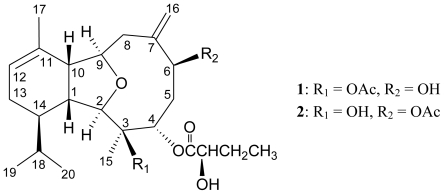
The structures of cladielloides A (**1**) and B (**2**).

Previous chemical investigations on the octocorals belonging to the genus *Cladiella* have resulted in a series of interesting eunicellin-based (2,11-cyclized cembranoid) diterpenoids [1–6], and the compounds of this type have been found to possess complex structures and various bioactivities [1–3,5–11]. In continuation of our search for bioactive substances from the marine invertebrates distributed in the tropical West Pacific Ocean, an Indonesian octocoral identified as *Cladiella* sp. was studied, and its organic extract exhibited cytotoxicity toward DLD-1 (human colorectal adenocarcinoma), HL-60 (human promyelocytic leukemia cells), and P388D1 (macrophage-like murine tumor cells), with IC_50_ = 2.7, 8.9, 7.2 μg/mL, respectively. Two new eunicellins, cladielloides A (**1**) and B (**2**), were isolated from this marine organism. In this paper, we report the isolation, structure determination, and bioactivity of the above new diterpenoids **1** and **2** (Scheme 1).

## 2. Results and Discussion

Cladielloide A (**1**) was isolated as a colorless oil and the molecular formula for this compound was determined to be C_26_H_40_O_7_ (seven degrees of unsaturation) by HRESIMS (C_26_H_40_O_7_ + Na, *m*/*z* 487.2674, calculated 487.2672). The IR spectrum of **1** showed bands at 3460 and 1734 cm^−1^, consistent with the presence of hydroxy and ester groups. From the ^1^H and ^13^C NMR spectra (Table 1), **1** was found to possess a trisubstituted olefin (*δ*_H_ 5.43, 1H, m, H-12; *δ*_C_ 132.1, s, C-11; 122.2, d, C-12), an exocyclic carbon-carbon double bond (*δ*_H_ 5.21, 1H, s, H-16a; 5.58, 1H, s, H-16b; *δ*_C_ 147.6, s, C-7; 115.2, t, C-16), an acetoxy group (*δ*_H_ 2.14, 3H, s; *δ*_C_ 20.6, q; 171.1, s), and a 2-hydroxybutyrate (*δ*_H_ 1.03, 3H, t, *J* = 7.2 Hz; 1.91, 2H, m; 4.86, 1H, dd, *J* = 6.8, 6.0 Hz; *δ*_C_ 9.3, q; 24.3, t; 74.1, d; 171.4, s) moiety. Thus, from the above data, four degrees of unsaturation were accounted for and compound **1** must be a tricyclic compound.

In the ^1^H NMR spectrum of **1**, two doublets at *δ*_H_ 0.92 and 0.83 (each 3H, d, *J* = 6.4 Hz, H_3_-19 and H_3_-20) were deduced from two methyls of an isopropyl group. A singlet of the tertiary methyl bonded to an oxygenated carbon was due to the resonance of signal at *δ*_H_ 1.37 (3H, s, H_3_-15). In addition, a suite of resonances of proton signals at *δ*_H_ 2.74 (1H, m, H-1), 2.63 (1H, br s, H-10), 3.86 (1H, d, *J* = 8.0 Hz, H-2), 4.16 (1H, dt, *J* = 3.6, 3.2 Hz, H-9), and carbon signals at *δ*_C_ 39.7 (d, C-1), 44.6 (d, C-10), 87.1 (d, C-2), and 81.3 (d, C-9), indicated the presence of a tetrahydrofuran structural unit. Based on the above data, the proposed skeleton of **1** was suggested to be a eunicellin-based metabolite.

From the ^1^H–^1^H COSY spectrum of **1**, it was possible to identify the separate spin systems among H-1/H-2; H-4/H_2_-5/H-6; H_2_-8/H-9/H-10; and H-10/H-1 (Table 1). These data, together with the HMBC correlations between H-1/C-2, −3, −10; H-2/C-3, −4, −9; H-4/C-3, −6; H_2_-5/C-3, −4, −6, −7; and H_2_-8/C-6, −7, −9, −10, established the connectivity from C-1 to C-10 within the ten-membered ring (Table 1). An exocyclic carbon-carbon double bond at C-7 was confirmed by the HMBC correlations between H_2_-16/C-6, −7, −8 and H_2_-8/C-16. The hydroxy proton signal at *δ*_H_ 2.84 was revealed by its ^1^H–^1^H COSY correlations to H-6 (*δ*_H_ 4.21), indicating its attachment to C-6. The location of 2-hydroxybutyrate group in **1** was confirmed by an HMBC correlation between H-4 (*δ*_H_ 5.14) and the 2-hydroxybutyrate carbonyl (*δ*_C_ 171.4, s). Thus, the remaining acetate ester was at C-3, an oxygenated quaternary carbon which bonded to the C-15 tertiary methyl and is confirmed by the HMBC correlations between H-2/C-15; H-4/C-15; and H_3_-15/C-2, −3, −4. The ether bridge between C-2 and C-9 was supported by an HMBC correlation between H-2/C-9. The 1-isopropyl-4-methylcyclohexene ring, which is fused to the ten-membered ring at C-1 and C-10, was elucidated by the ^1^H–^1^H COSY correlations between H-12/H_2_-13/H-14/H-1; H-14/H-18; and H-18/H_3_-19(H_3_-20) and further supported by the HMBC correlations between H-1/C-11, −14; H-2/C-14; H-10/C-11; H_3_-17/C-10; and H-18/C-1. The vinyl methyl at C-11 was confirmed by the HMBC correlations between H_3_-17/C-10, −11, −12 and further supported by the allylic coupling between the olefin proton H-12 and H_3_-17 (*J* = 0.8 Hz). Therefore, the planar structure of **1** was established.

The relative configuration of **1** was elucidated from the interactions observed in a NOESY experiment. In the NOESY experiment of **1** (Table 2), the correlations between H-1 with H-4 and H-10, indicated that these protons are situated on the same face and assigned as β protons. H-2 exhibited interactions with H-14 and H_3_-15 and no correlation was found between H-1 and H-2, indicating that H-2, H-14, and Me-15 should be α-oriented. H-6 correlated with one proton of C-5 methylene (*δ*_H_ 2.97), but not with H-4, reflecting the α-orientation of H-6. Furthermore, H-9 correlated with H_2_-8 and H_3_-17. From consideration of molecular models, H-9 was found to be reasonably close to H_2_-8 and H_3_-17, when H-9 was α-oriented in **1**.

**Figure 1:**
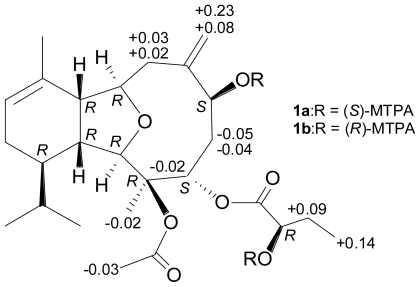
The key ^1^H NMR chemical shift differences Δ*δ* (*δ_S_*–*δ_R_*) in ppm for the MTPA esters of **1**.

In order to determine the absolute configuration, the eunicellin **1** was treated with (−) or (+)-MTPA chloride to yield the (*S*)- and (*R*)-MTPA esters **1a** and **1b**, respectively [12–14]. Comparison of the ^1^H NMR chemical shifts for **1a** and **1b** (Δ values shown in Figure 1) led to the assignment of the *S*-configuration at C-6. The C-24 hydroxy group in the 2-hydroxybutyrate moiety was also assigned as *R*-configuration. Therefore, the absolute configurations of all chiral centers of **1** were assigned as 1*R*, 2*R*, 3*R*, 4*S*, 6*S*, 9*R*, 10*R*, 14*R*, 24*R*.

Cladielloide B (**2**) had the same molecular formula as that of **1**, C_26_H_40_O_7_, as determined by HRESIMS (C_26_H_40_O_7_ + Na, *m*/*z* 487.2675, calculated 487.2672). The spectral data (1D, 2D NMR (Table 3), IR, and MS) were similar to those of **1**. However, the polarity of **2**, which was checked by TLC, was substantially different from that of **1**, indicating that these two compounds are isomers. In the ^1^H NMR spectrum of **2**, an acetate methyl was observed at *δ*_H_ 2.14 (3H, s). The additional acyl group was found to be a 2-hydroxybutyrate group, which showed six contiguous protons (*δ*_H_ 1.02, 3H, t, *J* = 7.2 Hz; 1.91, 2H, m; 4.87, 1H, dd, *J* = 6.8, 6.0 Hz). The ^13^C NMR signal at *δ*_C_ 170.2 (s) correlated with the signal of an oxymethine proton at *δ*_H_ 4.87 in the HMBC spectrum and was consequently assigned as the carbon atom of the 2-hydroxybutyrate carbonyl. A correlation observed in the HMBC experiment of **2** further revealed the connectivity between H-4 (*δ*_H_ 5.21) and the carbonyl carbon (*δ*_C_ 170.2) of 2-hydroxybutyrate unit and demonstrated the location of the 2-hydroxybutyrate to be at C-4. The position of acetoxy group at C-6 was also confirmed by the connectivity between the oxymethine proton at *δ*_H_ 4.66 (H-6) and the ester carbonyl at *δ*_C_ 171.6 (s) in the HMBC spectrum of **2**. Thus, the remaining hydroxy group should be positioned at C-3. In addition, by comparison of the NOESY correlations of **2** with those of **1**, the chiral centers of **2** were confirmed to be the same as those of **1**.

The cytotoxicity of metabolites **1** and **2** toward various tumor cell lines, including DLD-1, HL-60, CCRF-CEM (human T-cell acute lymphoblastic leukemia), and P388D1 cells was evaluated. The results, in Table 4, show that eunicellin **2** exhibited moderate cytotoxicity toward CCRF-CEM cells.

The *in vitro* anti-inflammatory effects of metabolites **1** and **2** were tested. Metabolite **2** displayed significant inhibitory effects on superoxide anion generation and elastase release by human neutrophils at 10 μg/mL (Table 5).

## 3. Experimental

### 3.1. General Experimental Procedures

Optical rotation values were measured with a JASCO P-1010 digital polarimeter at 25 °C. Infrared spectra were obtained on a VARIAN DIGLAB FTS 1000 FT-IR spectrometer. The NMR spectra were recorded on a VARIAN MERCURY PLUS 400 FT-NMR at 400 MHz for ^1^H and 100 MHz for ^13^C, in CDCl_3_ at 25 °C. Proton chemical shifts were referenced to the residual CHCl_3_ signal (*δ*_H_ 7.26 ppm). ^13^C NMR spectra were referenced to the center peak of CDCl_3_ at *δ*_C_ 77.1 ppm. ESIMS and HRESIMS data were recorded on a BRUKER APEX II mass spectrometer. Column chromatography was performed on silica gel (230–400 mesh, Merck, Darmstadt, Germany). TLC was carried out on precoated Kieselgel 60 F_254_ (0.25 mm, Merck) and spots were visualized by spraying with 10% H_2_SO_4_ solution followed by heating. HPLC was performed using a system comprised of a HITACHI L-7100 pump, a HITACHI photodiode array detector L-7455, and a RHEODYNE 7725 injection port. A normal phase column (Hibar 250 × 10 mm, Merck, silica gel 60, 5 μm,) was used for HPLC.

### 3.2. Animal Material

The octocoral *Cladiella* sp. were collected from Indonesia in 2004 and stored in a freezer until extraction. A voucher specimen was deposited in the National Museum of Marine Biology and Aquarium, Taiwan (NMMBA). This organism was identified by comparison with previous descriptions [15,16].

### 3.3. Extraction and Isolation

Slices of *Cladiella* sp. (wet weight 924 g) were extracted with a mixture of MeOH and CH_2_Cl_2_ (1:1) and the residue was partitioned between EtOAc and H_2_O. The EtOAc layer was subjected to silica gel column chromatography and eluted using a mixture of *n*-hexane and EtOAc (stepwise, 100:1 to pure EtOAc) to obtain 19 fractions A–S. Fractions K and N were repurified by normal phase HPLC, using the mixture of *n*-hexane/ethyl acetate to afford **2** (2.4 mg, 5.5:1) and **1** (7.9 mg, 3:1), respectively.

Cladielloide A (**1**). Colorless oil; [α]_D_^23^ −24° (*c* 0.4, CHCl_3_); IR (neat) ν_max_ 3460, 1734 cm^−1; 1^H (CDCl_3_, 400 MHz) and ^13^C (CDCl_3_, 100 MHz) NMR data, see Table 1; ESIMS *m*/*z* 487 (M + Na)^+^; HRESIMS *m*/*z* 487.2674 (calculated for C_26_H_40_O_7_ + Na, 487.2672).

Cladielloide B (**2**). Colorless oil; [α] _D_^23^ −10° (*c* 0.1, CHCl_3_); IR (neat) ν_max_ 3446, 1738 cm^−1; 1^H (CDCl_3_, 400 MHz) and ^13^C (CDCl_3_, 100 MHz) NMR data, see Table 3; ESIMS *m*/*z* 487 (M + Na)^+^; HRESIMS *m*/*z* 487.2675 (calculated for C_26_H_40_O_7_ + Na, 487.2672).

### 3.4. Preparation of (*S*)- and (*R*)-MTPA Esters of Cladielloide A (**1**)

To a solution of **1** (1 mg) in pyridine (0.4 mL), *R*-(−)-α-methoxy-α-(trifluoromethyl) phenylacetyl (MPTA) chloride (25 μL) was added, and the mixture was allowed to stand for 24 h at room temperature. The reaction was quenched by addition of 1.0 mL of water, and the mixture was subsequently extracted with EtOAc (3 × 1.0 mL). The EtOAc-soluble layers were combined, dried over anhydrous MgSO_4_ and evaporated. The residue was subjected to column chromatography over silica gel using *n*-hexane–EtOAc (13:2) to yield the (*S*)-MTPA ester, **1a** (0.7 mg, 44%). The same procedure was used to prepare the (*R*)-MTPA ester, **1b** (1.4 mg, 89%), from the reaction of (*S*)-MPTA chloride with **1** in pyridine. The key ^1^H NMR chemical shift differences Δ*δ* (*δ_S_* − *δ_R_*) in ppm for the MTPA esters of **1** are shown in Figure 1.

### 3.5. Cytotoxicity Testing

The cytotoxicity of compounds **1** and **2** was assayed with a modification of the MTT [3-(4,5-dimethylthiazol-2-yl)-2,5-diphenyltetrazolium bromide] colorimetric method. Cytotoxicity assays was carried out according to the procedures described previously [17,18].

### 3.6. Human Neutrophil Superoxide Anion Generation and Elastase Release

Human neutrophils were obtained by means of dextran sedimentation and Ficoll centrifugation. Superoxide generation and elastase release were carried out according to the procedures described previously [19,20]. Briefly, superoxide anion production was assayed by monitoring the superoxide dismutase-inhibitable reduction of ferricytochrome *c*. Elastase release experiments were performed using MeO-Suc-Ala-Ala-Pro-Valp-nitroanilide as the elastase substrate.

## Figures and Tables

**Table 1:** ^1^H and ^13^C NMR data, ^1^H–^1^H COSY, and HMBC correlations for diterpenoid **1**.

C/H	^1^H a	^13^C b		^1^H–^1^H COSY	HMBC (H→C)
1	2.74 m	39.7	(d) d	H-2, H-10, H-14	C-2, −3, −10, −11, −14
2	3.86 d (8.0) c	87.1	(d)	H-1	C-3, −4, −9, −14, −15
3		74.6	(s)		
4	5.14 dd (4.4, 3.6)	74.4	(d)	H_2_-5	C-3, −6, −15, −23
5α	2.97 ddd (16.0, 4.4, 2.8)	37.2	(t)	H-4, H-5β, H-6	C−3, −4
β	1.75 ddd (16.0, 5.6, 3.6)			H-4, H-5α, H-6	C-4, −6, −7
6	4.21 br s	72.6	(d)	H_2_-5, OH-6	n.o. e
7		147.6	(s)		
8	2.35 br d (2.4)	40.0	(t)	H-9	C-6, −7, −9, −10, −16
9	4.16 dt (3.6, 3.2)	81.3	(d)	H_2_-8, H-10	n.o.
10	2.63 br s	44.6	(d)	H-1, H-9	C-11
11		132.1	(s)		
12	5.43 m	122.2	(d)	H_2_-13, H_3_-17	n.o.
13α	2.10 m	22.8	(t)	H-12, H-13β, H-14	n.o.
β	1.97 m			H-12, H-13α, H-14	n.o.
14	1.58 m	39.0	(d)	H-1, H_2_-13, H-18	n.o.
15	1.37 s	22.4	(q)		C-2, −3, −4
16a	5.21 s	115.2	(t)	H-16b	C-6, −8
b	5.58 s			H-16a	C-6, −7, −8
17	1.68 d (0.8)	22.0	(q)	H-12	C-10, −11, −12
18	1.62 m	28.8	(d)	H-14, H_3_-19, H_3_-20	C-1, −14
19	0.92 d (6.4)	21.3	(q)	H-18	C-14, −18, −20
20	0.83 d (6.4)	20.5	(q)	H-18	C-14, −18, −19
OH-6	2.84 d (7.2)			H-6	n.o.
3-OC(O)CH_3_		171.1	(s)		
21 22	2.14 s	20.6	(q)		C-21
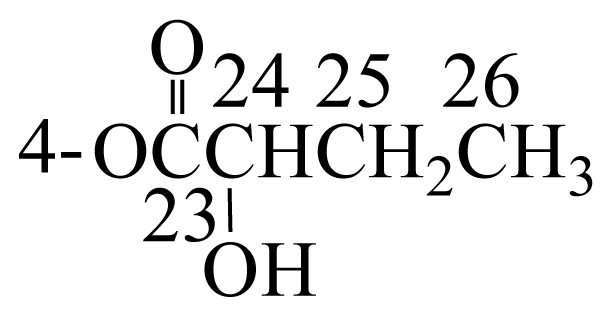		171.4	(s)		
	4.86 dd (6.8, 6.0)	74.1	(d)	H_2_-25	C-23, −25, −26
	1.91 m	24.3	(t)	H-24, H_3_-26	C-23, −24, −26
	1.03 t (7.2)	9.3	(q)	H_2_-25	C-24, −25

a Spectra measured at 400 MHz in CDCl_3_ at 25 °C;

b Spectra measured at 100 MHz in CDCl_3_ at 25 °C;

c *J* values (in hertz) in parentheses;

d Attached protons were deduced by DEPT and HMQC experiments;

e n.o. = not observed.

**Table 2:** The stereoview of **1** (generated from computer modeling) and the calculated distances (Å) between selected protons having key NOESY correlations.

Cladielloide A (1)	H/H	(Å)
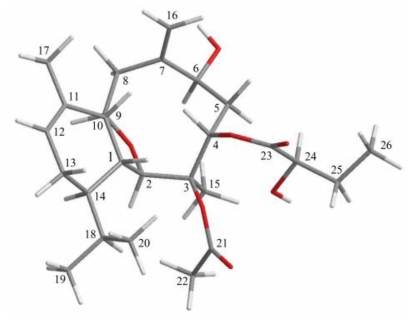	H-1/H-4	2.59
	H-1/H-10	2.33
	H-2/H-14	2.45
	H-2/H_3_-15	2.32
	H-6/H-5α	2.44
	H-8α/H-9	2.44
	H-8β/H-9	2.50
	H-9/H_3_-17	2.61

**Table 3:** ^1^H and ^13^C NMR data, ^1^H–^1^H COSY, and HMBC correlations for diterpenoid **2**.

Position	^1^H a	^13^C^b^		^1^H ^1^H COSY	HMBC (H→C)
1	2.51 m	40.6	(d) d	H-2, H-10, H-14	C-10
2	3.90 d (3.6) c	88.1	(d)	H-1	C-1, −3, −4
3		74.8	(s)		
4	5.21 dd (8.0, 4.0)	73.8	(d)	H_2_-5	C-6, −21
5α	2.48 m	34.2	(t)	H-4, H-5β, H-6	C-6, −7
β	1.97 m			H-4, H-5α, H-6	n.o. e
6	4.66 dd (8.8, 3.2)	83.8	(d)	H_2_-5	C-4, −7, −16, −25
7		144.2	(s)		
8α	2.65 dd (14.0, 4.8)	41.4	(t)	H-8β, H-9	C-6, −7, −9, −10, −16
β	2.46 dd (14.0, 2.0)			H-8α, H-9	C-6, −7, −9, −10, −16
9	4.06 br s	82.4	(d)	H_2_-8, H-10	n.o.
10	2.58 br s	44.7	(d)	H-1, H-9	C-8, −9, −11
11		131.1	(s)		
12	5.49 m	123.1	(d)	H_2_-13, H_3_-17	n.o.
13α	2.01 m	22.9	(t)	H-12, H-13β, H-14	n.o.
β	1.80 m			H-12, H-13α, H-14	n.o.
14	1.39 m	39.8	(d)	H-1, H_2_-13, H-18	C-2
15	1.33 s	22.8	(q)		C-2, −3, −4
16a	5.26 s	117.7	(t)	H-16b	C-6, −8
b	5.47 s			H-16a	C-6, −7, −8
17	1.69 d (1.2)	22.8	(q)	H-12	C-10, −11, −12
18	1.80 m	27.8	(d)	H-14, H_3_-19, H_3_-20	C-14, −19, −20
19	0.94 d (6.8)	21.7	(q)	H-18	C-14, −18, −20
20	0.77 d (6.8)	17.5	(q)	H-18	C-14, −19, −20
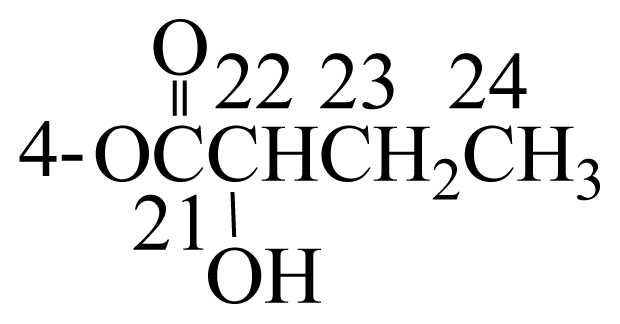		170.2	(s)		
	4.87 dd (6.8, 6.0)	74.3	(d)	H_2_-23	C-21, −23, −24
	1.91 m	24.5	(t)	H-22, H_3_-24	C-21, −22, −24
	1.02 t (7.2)	9.3	(q)	H_2_-23	C-22, −23
6-OC(O)CH_3_		171.6	(s)		
25 26	2.14 s	20.6	(q)		C-25

a Spectra measured at 400 MHz in CDCl_3_ at 25 °C;

b Spectra measured at 100 MHz in CDCl_3_ at 25 °C;

c *J* values (in hertz) in parentheses;

d Attached protons were deduced by DEPT and HMQC experiments;

e n.o. = not observed.

**Table 4:** Cytotoxic data of diterpenoids **1** and **2**.

Compound	Cell lines IC_50_ (μg/mL)			
	DLD-1	HL-60	CCRF-CEM	P388D1
**1**	>40	>40	>40	>40
**2**	10.2	>40	4.7	>40
Doxorubicin a	0.09	0.03	0.18	0.11

a Doxorubicin was used as a reference compound.

**Table 5:** Inhibitory effects of diterpenoids **1** and **2** on superoxide anion generation and elastase release by human neutrophils in response to FMLP/CB.

Compound	Superoxide anion	Elastase release

	IC_50_ (μg/mL) a or (Inh %) b	IC_50_ (μg/mL) or (Inh %)
**1**	(20.5 ± 5.0)	(27.1 ± 4.8)
**2**	5.9 ± 0.7	6.5 ± 1.9
DPI c	0.8 ± 0.2	
Elastatinal c		30.8 ± 5.7

a Concentration necessary for 50% inhibition (IC_50_);

b Percentage of inhibition (Inh %) at 10 μg/mL;

c DPI (diphenylene indonium) and elastatinal were used as reference compounds.
